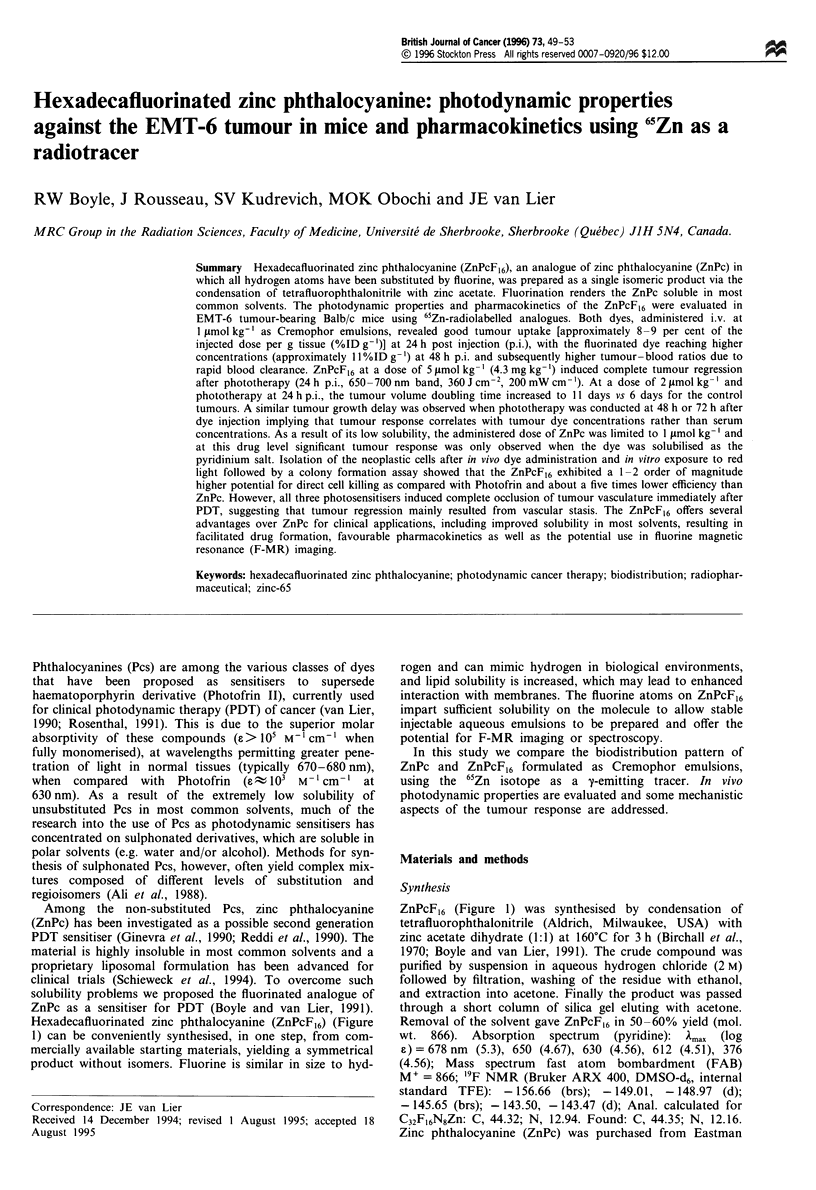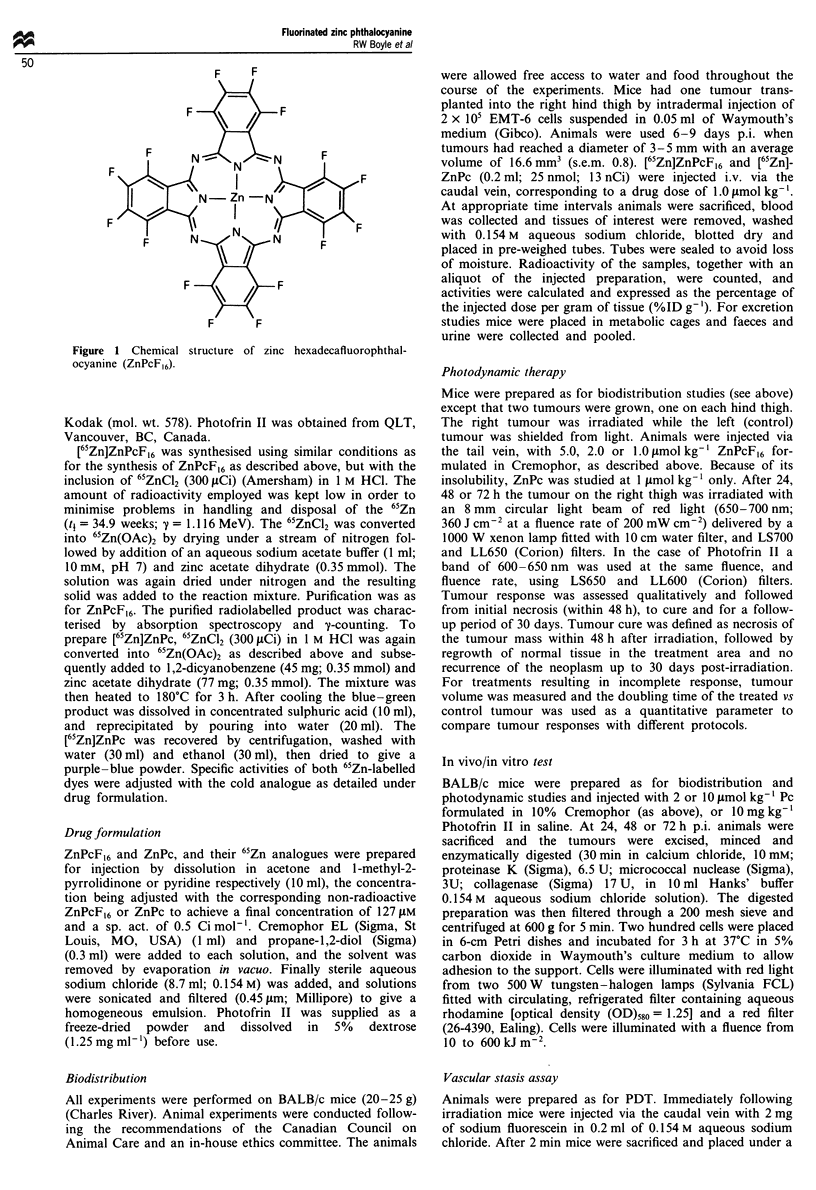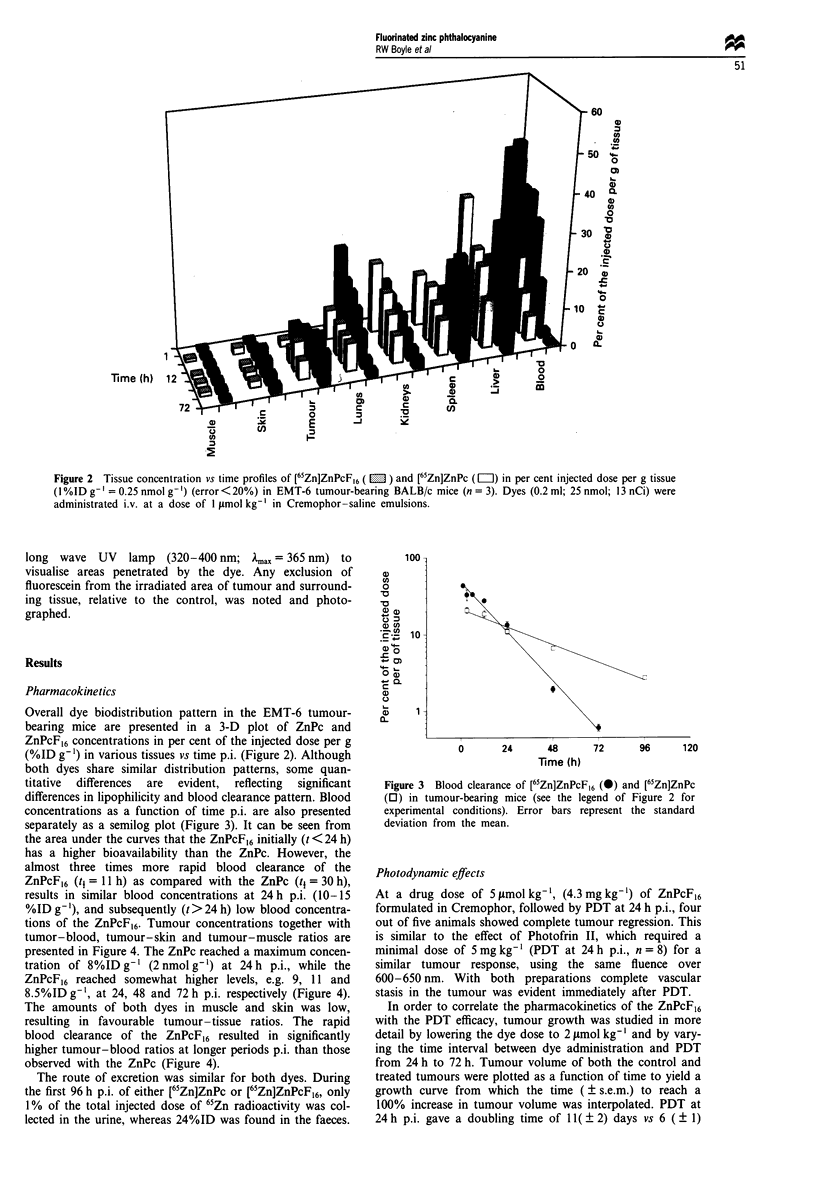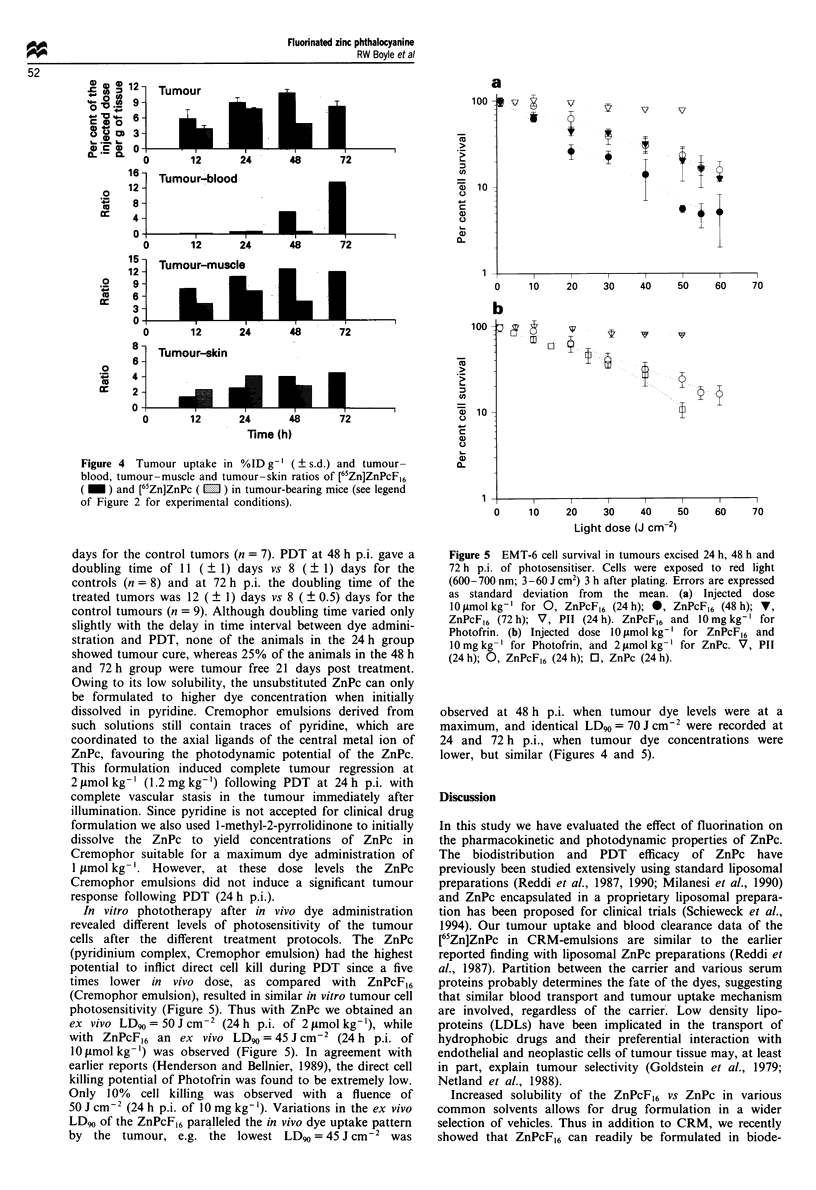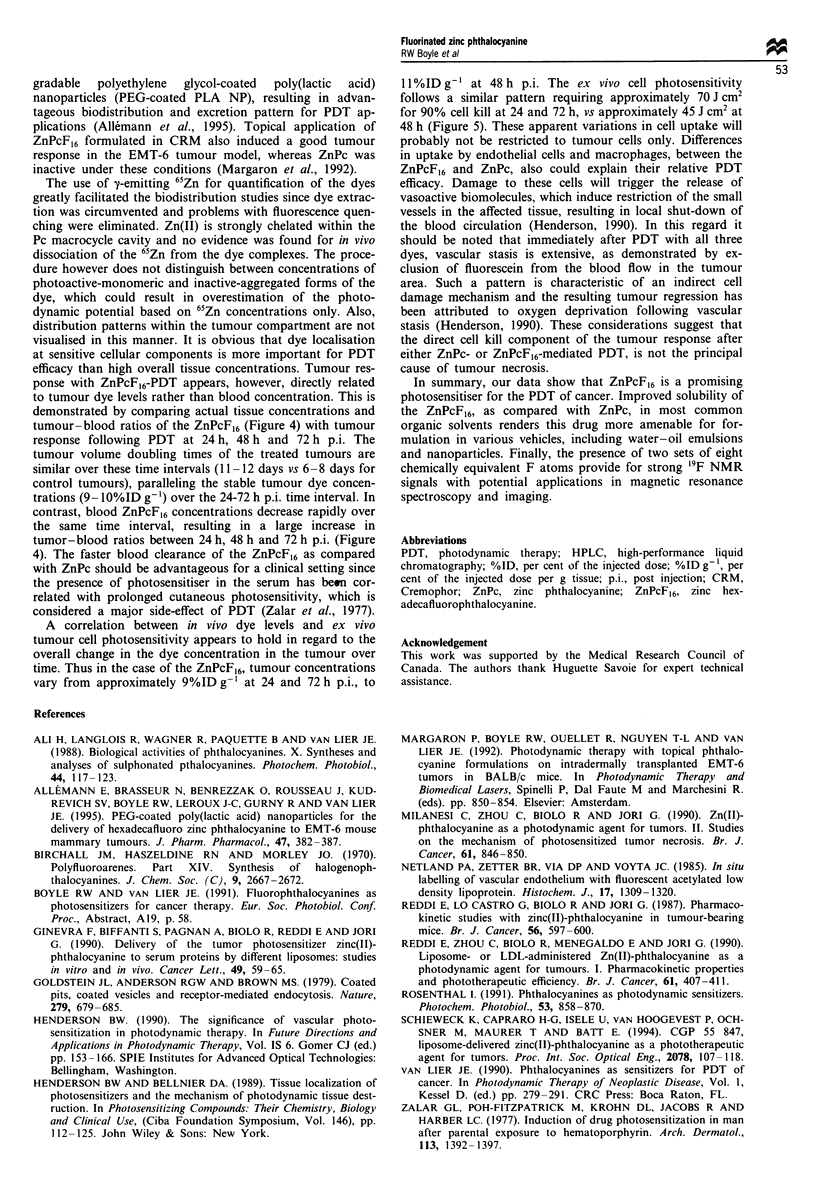# Hexadecafluorinated zinc phthalocyanine: photodynamic properties against the EMT-6 tumour in mice and pharmacokinetics using 65Zn as a radiotracer.

**DOI:** 10.1038/bjc.1996.9

**Published:** 1996-01

**Authors:** R. W. Boyle, J. Rousseau, S. V. Kudrevich, M. Obochi, J. E. van Lier

**Affiliations:** MRC Group in the Radiation Sciences, Faculty of Medicine, Université de Sherbrooke, Québec, Canada.

## Abstract

Hexadecafluorinated zinc phthalocyanine (ZnPcF16), an analogue of zinc phthalocyanine (ZnPc) in which all hydrogen atoms have been substituted by fluorine, was prepared as a single isomeric product via the condensation of tetrafluorophthalonitrile with zinc acetate. Fluorination renders the ZnPc soluble in most common solvents. The photodynamic properties and pharmacokinetics of the ZnPcF16 were evaluated in EMT-6 tumour-bearing Balb/c mice using 65Zn-radiolabelled analogues. Both dyes, administered i.v. at 1 mumol kg-1 as Cremophor emulsions, revealed good tumour uptake [approximately 8-9 per cent of the injected dose per g tissue (%IDg-1)] at 24 h post injection (p.i.), with the fluorinated dye reaching higher concentrations (approximately 11%IDg-1) at 48 h p.i. and subsequently higher tumour-blood ratios due to rapid blood clearance. ZnPcF16 at a dose of 5 mumol kg-1 (4.3 mg kg-1) induced complete tumour regression after phototherapy (24 h p.i., 650-700 nm band, 360 J cm-2, 200 mW cm-1). At a dose of 2 mumol kg-1 and phototherapy at 24 h p.i., the tumour volume doubling time increased to 11 days vs 6 days for the control tumours. A similar tumour growth delay was observed when phototherapy was conducted at 48 h or 72 h after dye injection implying that tumour response correlates with tumour dye concentrations rather than serum concentrations. As a result of its low solubility, the administered dose of ZnPc was limited to 1 mumol kg-1 and at this drug level significant tumour response was only observed when the dye was solubilised as the pyridinium salt. Isolation of the neoplastic cells after in vivo dye administration and in vitro exposure to red light followed by a colony formation assay showed that the ZnPcF16 exhibited a 1-2 order of magnitude higher potential for direct cell killing as compared with Photofrin and about a five times lower efficiency than ZnPc. However, all three photosensitisers induced complete occlusion of tumour vasculature immediately after PDT, suggesting that tumour regression mainly resulted from vascular stasis. The ZnPcF16 offers several advantages over ZnPc for clinical applications, including improved solubility in most solvents, resulting in facilitated drug formation, favourable pharmacokinetics as well as the potential use in fluorine magnetic resonance (F-MR) imaging.


British Journal of Cancer (1996) 73, 49–53
© 1996 Stockton Press All rights reserved 0007–0920/96 $12.00

### Hexadecafluorinated zinc phthalocyanine: photodynamic properties against the EMT-6 tumour in mice and pharmacokinetics using $^{65}Zn$ as a radiotracer

RW Boyle, J Rousseau, SV Kudrevich, MOK Obochi and JE van Lier

*MRC Group in the Radiation Sciences, Faculty of Medicine, Université de Sherbrooke, Sherbrooke (Québec) J1H 5N4, Canada.*

**Summary** Hexadecafluorinated zinc phthalocyanine ($ZnPcF_{16}$), an analogue of zinc phthalocyanine (ZnPc) in which all hydrogen atoms have been substituted by fluorine, was prepared as a single isomeric product via the condensation of tetrafluorophthalonitrile with zinc acetate. Fluorination renders the ZnPc soluble in most common solvents. The photodynamic properties and pharmacokinetics of the $ZnPcF_{16}$ were evaluated in EMT-6 tumour-bearing Balb/c mice using $^{65}Zn$-radiolabelled analogues. Both dyes, administered i.v. at 1 $\mu$mol kg$^{-1}$ as Cremophor emulsions, revealed good tumour uptake [approximately 8–9 per cent of the injected dose per g tissue (%ID g$^{-1}$)] at 24 h post injection (p.i.), with the fluorinated dye reaching higher concentrations (approximately 11%ID g$^{-1}$) at 48 h p.i. and subsequently higher tumour–blood ratios due to rapid blood clearance. $ZnPcF_{16}$ at a dose of 5 $\mu$mol kg$^{-1}$ (4.3 mg kg$^{-1}$) induced complete tumour regression after phototherapy (24 h p.i., 650–700 nm band, 360 J cm$^{-2}$, 200 mW cm$^{-1}$). At a dose of 2 $\mu$mol kg$^{-1}$ and phototherapy at 24 h p.i., the tumour volume doubling time increased to 11 days *vs* 6 days for the control tumours. A similar tumour growth delay was observed when phototherapy was conducted at 48 h or 72 h after dye injection implying that tumour response correlates with tumour dye concentrations rather than serum concentrations. As a result of its low solubility, the administered dose of ZnPc was limited to 1 $\mu$mol kg$^{-1}$ and at this drug level significant tumour response was only observed when the dye was solubilised as the pyridinium salt. Isolation of the neoplastic cells after *in vivo* dye administration and *in vitro* exposure to red light followed by a colony formation assay showed that the $ZnPcF_{16}$ exhibited a 1–2 order of magnitude higher potential for direct cell killing as compared with Photofrin and about a five times lower efficiency than ZnPc. However, all three photosensitisers induced complete occlusion of tumour vasculature immediately after PDT, suggesting that tumour regression mainly resulted from vascular stasis. The $ZnPcF_{16}$ offers several advantages over ZnPc for clinical applications, including improved solubility in most solvents, resulting in facilitated drug formation, favourable pharmacokinetics as well as the potential use in fluorine magnetic resonance (F-MR) imaging.

**Keywords:** hexadecafluorinated zinc phthalocyanine; photodynamic cancer therapy; biodistribution; radiopharmaceutical; zinc-65

Phthalocyanines (Pcs) are among the various classes of dyes that have been proposed as sensitisers to supersede haematoporphyrin derivative (Photofrin II), currently used for clinical photodynamic therapy (PDT) of cancer (van Lier, 1990; Rosenthal, 1991). This is due to the superior molar absorptivity of these compounds ($\varepsilon > 10^5$ M$^{-1}$ cm$^{-1}$ when fully monomerised), at wavelengths permitting greater penetration of light in normal tissues (typically 670–680 nm), when compared with Photofrin ($\varepsilon \approx 10^3$ M$^{-1}$ cm$^{-1}$ at 630 nm). As a result of the extremely low solubility of unsubstituted Pcs in most common solvents, much of the research into the use of Pcs as photodynamic sensitisers has concentrated on sulphonated derivatives, which are soluble in polar solvents (e.g. water and/or alcohol). Methods for synthesis of sulphonated Pcs, however, often yield complex mixtures composed of different levels of substitution and regioisomers (Ali *et al.*, 1988).

Among the non-substituted Pcs, zinc phthalocyanine (ZnPc) has been investigated as a possible second generation PDT sensitiser (Ginevra *et al.*, 1990; Reddi *et al.*, 1990). The material is highly insoluble in most common solvents and a proprietary liposomal formulation has been advanced for clinical trials (Schieweck *et al.*, 1994). To overcome such solubility problems we proposed the fluorinated analogue of ZnPc as a sensitiser for PDT (Boyle and van Lier, 1991). Hexadecafluorinated zinc phthalocyanine ($ZnPcF_{16}$) (Figure 1) can be conveniently synthesised, in one step, from commercially available starting materials, yielding a symmetrical product without isomers. Fluorine is similar in size to hydrogen and can mimic hydrogen in biological environments, and lipid solubility is increased, which may lead to enhanced interaction with membranes. The fluorine atoms on $ZnPcF_{16}$ impart sufficient solubility on the molecule to allow stable injectable aqueous emulsions to be prepared and offer the potential for F-MR imaging or spectroscopy.

In this study we compare the biodistribution pattern of ZnPc and $ZnPcF_{16}$ formulated as Cremophor emulsions, using the $^{65}Zn$ isotope as a $\gamma$-emitting tracer. *In vivo* photodynamic properties are evaluated and some mechanistic aspects of the tumour response are addressed.

### Materials and methods

### *Synthesis*

$ZnPcF_{16}$ (Figure 1) was synthesised by condensation of tetrafluorophthalonitrile (Aldrich, Milwaukee, USA) with zinc acetate dihydrate (1:1) at 160°C for 3 h (Birchall *et al.*, 1970; Boyle and van Lier, 1991). The crude compound was purified by suspension in aqueous hydrogen chloride (2 M) followed by filtration, washing of the residue with ethanol, and extraction into acetone. Finally the product was passed through a short column of silica gel eluting with acetone. Removal of the solvent gave $ZnPcF_{16}$ in 50–60% yield (mol. wt. 866). Absorption spectrum (pyridine): $\lambda_{max}$ (log $\varepsilon$) = 678 nm (5.3), 650 (4.67), 630 (4.56), 612 (4.51), 376 (4.56); Mass spectrum fast atom bombardment (FAB) $M^+ = 866$; $^{19}F$ NMR (Bruker ARX 400, DMSO-$d_6$, internal standard TFE): −156.66 (brs); −149.01, −148.97 (d); −145.65 (brs); −143.50, −143.47 (d); Anal. calculated for $C_{32}F_{16}N_8Zn$: C, 44.32; N, 12.94. Found: C, 44.35; N, 12.16. Zinc phthalocyanine (ZnPc) was purchased from Eastman

Correspondence: JE van Lier
Received 14 December 1994; revised 1 August 1995; accepted 18 August 1995

**Figure 1** Chemical structure of zinc hexadecafluorophthalocyanine ($ZnPcF_{16}$).

Kodak (mol. wt. 578). Photofrin II was obtained from QLT, Vancouver, BC, Canada.

[$^{65}$Zn]$ZnPcF_{16}$ was synthesised using similar conditions as for the synthesis of $ZnPcF_{16}$ as described above, but with the inclusion of $^{65}ZnCl_2$ (300 μCi) (Amersham) in 1 M HCl. The amount of radioactivity employed was kept low in order to minimise problems in handling and disposal of the $^{65}$Zn ($t_{\frac{1}{2}}$ = 34.9 weeks; γ = 1.116 MeV). The $^{65}ZnCl_2$ was converted into $^{65}Zn(OAc)_2$ by drying under a stream of nitrogen followed by addition of an aqueous sodium acetate buffer (1 ml; 10 mM, pH 7) and zinc acetate dihydrate (0.35 mmol). The solution was again dried under nitrogen and the resulting solid was added to the reaction mixture. Purification was as for $ZnPcF_{16}$. The purified radiolabelled product was characterised by absorption spectroscopy and γ-counting. To prepare [$^{65}$Zn]ZnPc, $^{65}ZnCl_2$ (300 μCi) in 1 M HCl was again converted into $^{65}Zn(OAc)_2$ as described above and subsequently added to 1,2-dicyanobenzene (45 mg; 0.35 mmol) and zinc acetate dihydrate (77 mg; 0.35 mmol). The mixture was then heated to 180°C for 3 h. After cooling the blue–green product was dissolved in concentrated sulphuric acid (10 ml), and reprecipitated by pouring into water (20 ml). The [$^{65}$Zn]ZnPc was recovered by centrifugation, washed with water (30 ml) and ethanol (30 ml), then dried to give a purple–blue powder. Specific activities of both $^{65}$Zn-labelled dyes were adjusted with the cold analogue as detailed under drug formulation.

*Drug formulation*

$ZnPcF_{16}$ and ZnPc, and their $^{65}$Zn analogues were prepared for injection by dissolution in acetone and 1-methyl-2-pyrrolidinone or pyridine respectively (10 ml), the concentration being adjusted with the corresponding non-radioactive $ZnPcF_{16}$ or ZnPc to achieve a final concentration of 127 μM and a sp. act. of 0.5 Ci $mol^{-1}$. Cremophor EL (Sigma, St Louis, MO, USA) (1 ml) and propane-1,2-diol (Sigma) (0.3 ml) were added to each solution, and the solvent was removed by evaporation *in vacuo*. Finally sterile aqueous sodium chloride (8.7 ml; 0.154 M) was added, and solutions were sonicated and filtered (0.45 μm; Millipore) to give a homogeneous emulsion. Photofrin II was supplied as a freeze-dried powder and dissolved in 5% dextrose (1.25 mg $ml^{-1}$) before use.

*Biodistribution*

All experiments were performed on BALB/c mice (20–25 g) (Charles River). Animal experiments were conducted following the recommendations of the Canadian Council on Animal Care and an in-house ethics committee. The animals were allowed free access to water and food throughout the course of the experiments. Mice had one tumour transplanted into the right hind thigh by intradermal injection of $2 \times 10^5$ EMT-6 cells suspended in 0.05 ml of Waymouth's medium (Gibco). Animals were used 6–9 days p.i. when tumours had reached a diameter of 3–5 mm with an average volume of 16.6 $mm^3$ (s.e.m. 0.8). [$^{65}$Zn]$ZnPcF_{16}$ and [$^{65}$Zn]-ZnPc (0.2 ml; 25 nmol; 13 nCi) were injected i.v. via the caudal vein, corresponding to a drug dose of 1.0 μmol $kg^{-1}$. At appropriate time intervals animals were sacrificed, blood was collected and tissues of interest were removed, washed with 0.154 M aqueous sodium chloride, blotted dry and placed in pre-weighed tubes. Tubes were sealed to avoid loss of moisture. Radioactivity of the samples, together with an aliquot of the injected preparation, were counted, and activities were calculated and expressed as the percentage of the injected dose per gram of tissue (%ID $g^{-1}$). For excretion studies mice were placed in metabolic cages and faeces and urine were collected and pooled.

*Photodynamic therapy*

Mice were prepared as for biodistribution studies (see above) except that two tumours were grown, one on each hind thigh. The right tumour was irradiated while the left (control) tumour was shielded from light. Animals were injected via the tail vein, with 5.0, 2.0 or 1.0 μmol $kg^{-1}$ $ZnPcF_{16}$ formulated in Cremophor, as described above. Because of its insolubility, ZnPc was studied at 1 μmol $kg^{-1}$ only. After 24, 48 or 72 h the tumour on the right thigh was irradiated with an 8 mm circular light beam of red light (650–700 nm; 360 J $cm^{-2}$ at a fluence rate of 200 mW $cm^{-2}$) delivered by a 1000 W xenon lamp fitted with 10 cm water filter, and LS700 and LL650 (Corion) filters. In the case of Photofrin II a band of 600–650 nm was used at the same fluence, and fluence rate, using LS650 and LL600 (Corion) filters. Tumour response was assessed qualitatively and followed from initial necrosis (within 48 h), to cure and for a follow-up period of 30 days. Tumour cure was defined as necrosis of the tumour mass within 48 h after irradiation, followed by regrowth of normal tissue in the treatment area and no recurrence of the neoplasm up to 30 days post-irradiation. For treatments resulting in incomplete response, tumour volume was measured and the doubling time of the treated *vs* control tumour was used as a quantitative parameter to compare tumour responses with different protocols.

In vivo/in vitro *test*

BALB/c mice were prepared as for biodistribution and photodynamic studies and injected with 2 or 10 μmol $kg^{-1}$ Pc formulated in 10% Cremophor (as above), or 10 mg $kg^{-1}$ Photofrin II in saline. At 24, 48 or 72 h p.i. animals were sacrificed and the tumours were excised, minced and enzymatically digested (30 min in calcium chloride, 10 mM; proteinase K (Sigma), 6.5 U; micrococcal nuclease (Sigma), 3U; collagenase (Sigma) 17 U, in 10 ml Hanks' buffer 0.154 M aqueous sodium chloride solution). The digested preparation was then filtered through a 200 mesh sieve and centrifuged at 600 g for 5 min. Two hundred cells were placed in 6-cm Petri dishes and incubated for 3 h at 37°C in 5% carbon dioxide in Waymouth's culture medium to allow adhesion to the support. Cells were illuminated with red light from two 500 W tungsten–halogen lamps (Sylvania FCL) fitted with circulating, refrigerated filter containing aqueous rhodamine [optical density $(OD)_{580} = 1.25$] and a red filter (26-4390, Ealing). Cells were illuminated with a fluence from 10 to 600 kJ $m^{-2}$.

*Vascular stasis assay*

Animals were prepared as for PDT. Immediately following irradiation mice were injected via the caudal vein with 2 mg of sodium fluorescein in 0.2 ml of 0.154 M aqueous sodium chloride. After 2 min mice were sacrificed and placed under a

**Figure 2** Tissue concentration *vs* time profiles of [$^{65}$Zn]ZnPcF$_{16}$ ( ▩ ) and [$^{65}$Zn]ZnPc (□) in per cent injected dose per g tissue (1%ID g$^{-1}$ = 0.25 nmol g$^{-1}$) (error < 20%) in EMT-6 tumour-bearing BALB/c mice ($n$ = 3). Dyes (0.2 ml; 25 nmol; 13 nCi) were administrated i.v. at a dose of 1 μmol kg$^{-1}$ in Cremophor–saline emulsions.

long wave UV lamp (320–400 nm; $\lambda_{max}$ = 365 nm) to visualise areas penetrated by the dye. Any exclusion of fluorescein from the irradiated area of tumour and surrounding tissue, relative to the control, was noted and photographed.

### Results

### *Pharmacokinetics*

Overall dye biodistribution pattern in the EMT-6 tumour-bearing mice are presented in a 3-D plot of ZnPc and ZnPcF$_{16}$ concentrations in per cent of the injected dose per g (%ID g$^{-1}$) in various tissues *vs* time p.i. (Figure 2). Although both dyes share similar distribution patterns, some quantitative differences are evident, reflecting significant differences in lipophilicity and blood clearance pattern. Blood concentrations as a function of time p.i. are also presented separately as a semilog plot (Figure 3). It can be seen from the area under the curves that the ZnPcF$_{16}$ initially ($t$ < 24 h) has a higher bioavailability than the ZnPc. However, the almost three times more rapid blood clearance of the ZnPcF$_{16}$ ($t_{½}$ = 11 h) as compared with the ZnPc ($t_{½}$ = 30 h), results in similar blood concentrations at 24 h p.i. (10–15 %ID g$^{-1}$), and subsequently ($t$ > 24 h) low blood concentrations of the ZnPcF$_{16}$. Tumour concentrations together with tumor–blood, tumour–skin and tumour–muscle ratios are presented in Figure 4. The ZnPc reached a maximum concentration of 8%ID g$^{-1}$ (2 nmol g$^{-1}$) at 24 h p.i., while the ZnPcF$_{16}$ reached somewhat higher levels, e.g. 9, 11 and 8.5%ID g$^{-1}$, at 24, 48 and 72 h p.i. respectively (Figure 4). The amounts of both dyes in muscle and skin was low, resulting in favourable tumour–tissue ratios. The rapid blood clearance of the ZnPcF$_{16}$ resulted in significantly higher tumour–blood ratios at longer periods p.i. than those observed with the ZnPc (Figure 4).

The route of excretion was similar for both dyes. During the first 96 h p.i. of either [$^{65}$Zn]ZnPc or [$^{65}$Zn]ZnPcF$_{16}$, only 1% of the total injected dose of $^{65}$Zn radioactivity was collected in the urine, whereas 24%ID was found in the faeces.

**Figure 3** Blood clearance of [$^{65}$Zn]ZnPcF$_{16}$ (●) and [$^{65}$Zn]ZnPc (□) in tumour-bearing mice (see the legend of Figure 2 for experimental conditions). Error bars represent the standard deviation from the mean.

### *Photodynamic effects*

At a drug dose of 5 μmol kg$^{-1}$, (4.3 mg kg$^{-1}$) of ZnPcF$_{16}$ formulated in Cremophor, followed by PDT at 24 h p.i., four out of five animals showed complete tumour regression. This is similar to the effect of Photofrin II, which required a minimal dose of 5 mg kg$^{-1}$ (PDT at 24 h p.i., $n$ = 8) for a similar tumour response, using the same fluence over 600–650 nm. With both preparations complete vascular stasis in the tumour was evident immediately after PDT.

In order to correlate the pharmacokinetics of the ZnPcF$_{16}$ with the PDT efficacy, tumour growth was studied in more detail by lowering the dye dose to 2 μmol kg$^{-1}$ and by varying the time interval between dye administration and PDT from 24 h to 72 h. Tumour volume of both the control and treated tumours were plotted as a function of time to yield a growth curve from which the time (± s.e.m.) to reach a 100% increase in tumour volume was interpolated. PDT at 24 h p.i. gave a doubling time of 11(±2) days *vs* 6 (±1)

**Figure 4** Tumour uptake in %ID $g^{-1}$ ($\pm$s.d.) and tumour–blood, tumour–muscle and tumour–skin ratios of [$^{65}$Zn]ZnPcF$_{16}$ (■) and [$^{65}$Zn]ZnPc (▒) in tumour-bearing mice (see legend of Figure 2 for experimental conditions).

**Figure 5** EMT-6 cell survival in tumours excised 24 h, 48 h and 72 h p.i. of photosensitiser. Cells were exposed to red light (600–700 nm; 3–60 J cm$^2$) 3 h after plating. Errors are expressed as standard deviation from the mean. (**a**) Injected dose 10 μmol $kg^{-1}$ for O, ZnPcF$_{16}$ (24 h); ●, ZnPcF$_{16}$ (48 h); ▼, ZnPcF$_{16}$ (72 h); ∇, PII (24 h). ZnPcF$_{16}$ and 10 mg $kg^{-1}$ for Photofrin. (**b**) Injected dose 10 μmol $kg^{-1}$ for ZnPcF$_{16}$ and 10 mg $kg^{-1}$ for Photofrin, and 2 μmol $kg^{-1}$ for ZnPc. ∇, PII (24 h); O, ZnPcF$_{16}$ (24 h); □, ZnPc (24 h).

days for the control tumors ($n = 7$). PDT at 48 h p.i. gave a doubling time of 11 ($\pm$1) days *vs* 8 ($\pm$1) days for the controls ($n = 8$) and at 72 h p.i. the doubling time of the treated tumors was 12 ($\pm$1) days *vs* 8 ($\pm$0.5) days for the control tumours ($n = 9$). Although doubling time varied only slightly with the delay in time interval between dye administration and PDT, none of the animals in the 24 h group showed tumour cure, whereas 25% of the animals in the 48 h and 72 h group were tumour free 21 days post treatment. Owing to its low solubility, the unsubstituted ZnPc can only be formulated to higher dye concentration when initially dissolved in pyridine. Cremophor emulsions derived from such solutions still contain traces of pyridine, which are coordinated to the axial ligands of the central metal ion of ZnPc, favouring the photodynamic potential of the ZnPc. This formulation induced complete tumour regression at 2 μmol $kg^{-1}$ (1.2 mg $kg^{-1}$) following PDT at 24 h p.i. with complete vascular stasis in the tumour immediately after illumination. Since pyridine is not accepted for clinical drug formulation we also used 1-methyl-2-pyrrolidinone to initially dissolve the ZnPc to yield concentrations of ZnPc in Cremophor suitable for a maximum dye administration of 1 μmol $kg^{-1}$. However, at these dose levels the ZnPc Cremophor emulsions did not induce a significant tumour response following PDT (24 h p.i.).

*In vitro* phototherapy after *in vivo* dye administration revealed different levels of photosensitivity of the tumour cells after the different treatment protocols. The ZnPc (pyridinium complex, Cremophor emulsion) had the highest potential to inflict direct cell kill during PDT since a five times lower *in vivo* dose, as compared with ZnPcF$_{16}$ (Cremophor emulsion), resulted in similar *in vitro* tumour cell photosensitivity (Figure 5). Thus with ZnPc we obtained an *ex vivo* $LD_{90} = 50$ J $cm^{-2}$ (24 h p.i. of 2 μmol $kg^{-1}$), while with ZnPcF$_{16}$ an *ex vivo* $LD_{90} = 45$ J $cm^{-2}$ (24 h p.i. of 10 μmol $kg^{-1}$) was observed (Figure 5). In agreement with earlier reports (Henderson and Bellnier, 1989), the direct cell killing potential of Photofrin was found to be extremely low. Only 10% cell killing was observed with a fluence of 50 J $cm^{-2}$ (24 h p.i. of 10 mg $kg^{-1}$). Variations in the *ex vivo* $LD_{90}$ of the ZnPcF$_{16}$ paralleled the *in vivo* dye uptake pattern by the tumour, e.g. the lowest $LD_{90} = 45$ J $cm^{-2}$ was observed at 48 h p.i. when tumour dye levels were at a maximum, and identical $LD_{90} = 70$ J $cm^{-2}$ were recorded at 24 and 72 h p.i., when tumour dye concentrations were lower, but similar (Figures 4 and 5).

### Discussion

In this study we have evaluated the effect of fluorination on the pharmacokinetic and photodynamic properties of ZnPc. The biodistribution and PDT efficacy of ZnPc have previously been studied extensively using standard liposomal preparations (Reddi *et al.*, 1987, 1990; Milanesi *et al.*, 1990) and ZnPc encapsulated in a proprietary liposomal preparation has been proposed for clinical trials (Schieweck *et al.*, 1994). Our tumour uptake and blood clearance data of the [$^{65}$Zn]ZnPc in CRM-emulsions are similar to the earlier reported finding with liposomal ZnPc preparations (Reddi *et al.*, 1987). Partition between the carrier and various serum proteins probably determines the fate of the dyes, suggesting that similar blood transport and tumour uptake mechanism are involved, regardless of the carrier. Low density lipoproteins (LDLs) have been implicated in the transport of hydrophobic drugs and their preferential interaction with endothelial and neoplastic cells of tumour tissue may, at least in part, explain tumour selectivity (Goldstein *et al.*, 1979; Netland *et al.*, 1988).

Increased solubility of the ZnPcF$_{16}$ *vs* ZnPc in various common solvents allows for drug formulation in a wider selection of vehicles. Thus in addition to CRM, we recently showed that ZnPcF$_{16}$ can readily be formulated in biodegradable polyethylene glycol-coated poly(lactic acid) nanoparticles (PEG-coated PLA NP), resulting in advantageous biodistribution and excretion pattern for PDT applications (Allémann *et al.*, 1995). Topical application of $ZnPcF_{16}$ formulated in CRM also induced a good tumour response in the EMT-6 tumour model, whereas ZnPc was inactive under these conditions (Margaron *et al.*, 1992).

The use of γ-emitting $^{65}Zn$ for quantification of the dyes greatly facilitated the biodistribution studies since dye extraction was circumvented and problems with fluorescence quenching were eliminated. Zn(II) is strongly chelated within the Pc macrocycle cavity and no evidence was found for *in vivo* dissociation of the $^{65}Zn$ from the dye complexes. The procedure however does not distinguish between concentrations of photoactive-monomeric and inactive-aggregated forms of the dye, which could result in overestimation of the photodynamic potential based on $^{65}Zn$ concentrations only. Also, distribution patterns within the tumour compartment are not visualised in this manner. It is obvious that dye localisation at sensitive cellular components is more important for PDT efficacy than high overall tissue concentrations. Tumour response with $ZnPcF_{16}$-PDT appears, however, directly related to tumour dye levels rather than blood concentration. This is demonstrated by comparing actual tissue concentrations and tumour–blood ratios of the $ZnPcF_{16}$ (Figure 4) with tumour response following PDT at 24 h, 48 h and 72 h p.i. The tumour volume doubling times of the treated tumours are similar over these time intervals (11–12 days *vs* 6–8 days for control tumours), paralleling the stable tumour dye concentrations (9–10%ID $g^{-1}$) over the 24-72 h p.i. time interval. In contrast, blood $ZnPcF_{16}$ concentrations decrease rapidly over the same time interval, resulting in a large increase in tumor–blood ratios between 24 h, 48 h and 72 h p.i. (Figure 4). The faster blood clearance of the $ZnPcF_{16}$ as compared with ZnPc should be advantageous for a clinical setting since the presence of photosensitiser in the serum has been correlated with prolonged cutaneous photosensitivity, which is considered a major side-effect of PDT (Zalar *et al.*, 1977).

A correlation between *in vivo* dye levels and *ex vivo* tumour cell photosensitivity appears to hold in regard to the overall change in the dye concentration in the tumour over time. Thus in the case of the $ZnPcF_{16}$, tumour concentrations vary from approximately 9%ID $g^{-1}$ at 24 and 72 h p.i., to 11%ID $g^{-1}$ at 48 h p.i. The *ex vivo* cell photosensitivity follows a similar pattern requiring approximately 70 J $cm^2$ for 90% cell kill at 24 and 72 h, *vs* approximately 45 J $cm^2$ at 48 h (Figure 5). These apparent variations in cell uptake will probably not be restricted to tumour cells only. Differences in uptake by endothelial cells and macrophages, between the $ZnPcF_{16}$ and ZnPc, also could explain their relative PDT efficacy. Damage to these cells will trigger the release of vasoactive biomolecules, which induce restriction of the small vessels in the affected tissue, resulting in local shut-down of the blood circulation (Henderson, 1990). In this regard it should be noted that immediately after PDT with all three dyes, vascular stasis is extensive, as demonstrated by exclusion of fluorescein from the blood flow in the tumour area. Such a pattern is characteristic of an indirect cell damage mechanism and the resulting tumour regression has been attributed to oxygen deprivation following vascular stasis (Henderson, 1990). These considerations suggest that the direct cell kill component of the tumour response after either ZnPc- or $ZnPcF_{16}$-mediated PDT, is not the principal cause of tumour necrosis.

In summary, our data show that $ZnPcF_{16}$ is a promising photosensitiser for the PDT of cancer. Improved solubility of the $ZnPcF_{16}$, as compared with ZnPc, in most common organic solvents renders this drug more amenable for formulation in various vehicles, including water–oil emulsions and nanoparticles. Finally, the presence of two sets of eight chemically equivalent F atoms provide for strong $^{19}F$ NMR signals with potential applications in magnetic resonance spectroscopy and imaging.

### Abbreviations

PDT, photodynamic therapy; HPLC, high-performance liquid chromatography; %ID, per cent of the injected dose; %ID $g^{-1}$, per cent of the injected dose per g tissue; p.i., post injection; CRM, Cremophor; ZnPc, zinc phthalocyanine; $ZnPcF_{16}$, zinc hexadecafluorophthalocyanine.

### Acknowledgement

This work was supported by the Medical Research Council of Canada. The authors thank Huguette Savoie for expert technical assistance.

### References

ALI H, LANGLOIS R, WAGNER R, PAQUETTE B AND VAN LIER JE. (1988). Biological activities of phthalocyanines. X. Syntheses and analyses of sulphonated pthalocyanines. *Photochem. Photobiol.*, **44**, 117–123.

ALLÉMANN E, BRASSEUR N, BENREZZAK O, ROUSSEAU J, KUDREVICH SV, BOYLE RW, LEROUX J-C, GURNY R AND VAN LIER JE. (1995). PEG-coated poly(lactic acid) nanoparticles for the delivery of hexadecafluoro zinc phthalocyanine to EMT-6 mouse mammary tumours. *J. Pharm. Pharmacol.*, **47**, 382–387.

BIRCHALL JM, HASZELDINE RN AND MORLEY JO. (1970). Polyfluoroarenes. Part XIV. Synthesis of halogenophthalocyanines. *J. Chem. Soc. (C)*, **9**, 2667–2672.

BOYLE RW AND VAN LIER JE. (1991). Fluorophthalocyanines as photosensitizers for cancer therapy. *Eur. Soc. Photobiol. Conf. Proc.*, Abstract, A19, p. 58.

GINEVRA F, BIFFANTI S, PAGNAN A, BIOLO R, REDDI E AND JORI G. (1990). Delivery of the tumor photosensitizer zinc(II)-phthalocyanine to serum proteins by different liposomes: studies *in vitro* and *in vivo*. *Cancer Lett.*, **49**, 59–65.

GOLDSTEIN JL, ANDERSON RGW AND BROWN MS. (1979). Coated pits, coated vesicles and receptor-mediated endocytosis. *Nature*, **279**, 679–685.

HENDERSON BW. (1990). The significance of vascular photosensitization in photodynamic therapy. In *Future Directions and Applications in Photodynamic Therapy*, Vol. IS 6. Gomer CJ (ed.) pp. 153–166. SPIE Institutes for Advanced Optical Technologies: Bellingham, Washington.

HENDERSON BW AND BELLNIER DA. (1989). Tissue localization of photosensitizers and the mechanism of photodynamic tissue destruction. In *Photosensitizing Compounds: Their Chemistry, Biology and Clinical Use*, (Ciba Foundation Symposium, Vol. 146), pp. 112–125. John Wiley & Sons: New York.

MARGARON P, BOYLE RW, OUELLET R, NGUYEN T-L AND VAN LIER JE. (1992). Photodynamic therapy with topical phthalocyanine formulations on intradermally transplanted EMT-6 tumors in BALB/c mice. In *Photodynamic Therapy and Biomedical Lasers*, Spinelli P, Dal Faute M and Marchesini R. (eds). pp. 850–854. Elsevier: Amsterdam.

MILANESI C, ZHOU C, BIOLO R AND JORI G. (1990). Zn(II)-phthalocyanine as a photodynamic agent for tumors. II. Studies on the mechanism of photosensitized tumor necrosis. *Br. J. Cancer*, **61**, 846–850.

NETLAND PA, ZETTER BR, VIA DP AND VOYTA JC. (1985). *In situ* labelling of vascular endothelium with fluorescent acetylated low density lipoprotein. *Histochem. J.*, **17**, 1309–1320.

REDDI E, LO CASTRO G, BIOLO R AND JORI G. (1987). Pharmacokinetic studies with zinc(II)-phthalocyanine in tumour-bearing mice. *Br. J. Cancer*, **56**, 597–600.

REDDI E, ZHOU C, BIOLO R, MENEGALDO E AND JORI G. (1990). Liposome- or LDL-administered Zn(II)-phthalocyanine as a photodynamic agent for tumours. I. Pharmacokinetic properties and phototherapeutic efficiency. *Br. J. Cancer*, **61**, 407–411.

ROSENTHAL I. (1991). Phthalocyanines as photodynamic sensitizers. *Photochem. Photobiol.*, **53**, 858–870.

SCHIEWECK K, CAPRARO H-G, ISELE U, VAN HOOGEVEST P, OCHSNER M, MAURER T AND BATT E. (1994). CGP 55 847, liposome-delivered zinc(II)-phthalocyanine as a phototherapeutic agent for tumors. *Proc. Int. Soc. Optical Eng.*, **2078**, 107–118.

VAN LIER JE. (1990). Phthalocyanines as sensitizers for PDT of cancer. In *Photodynamic Therapy of Neoplastic Disease*, Vol. 1, Kessel D. (ed.) pp. 279–291. CRC Press: Boca Raton, FL.

ZALAR GL, POH-FITZPATRICK M, KROHN DL, JACOBS R AND HARBER LC. (1977). Induction of drug photosensitization in man after parental exposure to hematoporphyrin. *Arch. Dermatol.*, **113**, 1392–1397.